# Photocoagulation of Human Retinal Pigment Epithelial Cells *In Vitro*: Evaluation of Necrosis, Apoptosis, Cell Migration, Cell Proliferation and Expression of Tissue Repairing and Cytoprotective Genes

**DOI:** 10.1371/journal.pone.0070465

**Published:** 2013-08-01

**Authors:** Poya Tababat-Khani, Lisa M. Berglund, Carl-David Agardh, Maria F. Gomez, Elisabet Agardh

**Affiliations:** 1 Unit on Vascular Diabetic Complications, Ophthalmology, Department of Clinical Sciences Malmö, Lund University, Malmö, Sweden; 2 Vascular Excitation-Transcription Coupling, Department of Clinical Sciences Malmö, Lund University, Malmö, Sweden; University of Florida, United States of America

## Abstract

**Aims:**

Sight-threatening diabetic retinopathy has been treated with photocoagulation for decades but the mechanisms behind the beneficial clinical effects are poorly understood. One target of irradiation and a potential player in this process is the retinal pigment epithelium (RPE). Here we establish an in vitro model for photocoagulation of human RPE cells.

**Methods:**

ARPE-19 cells were exposed to photocoagulation and studied at various time points up to 168h. Lesion morphology, necrosis and apoptosis were investigated by light microscopy; LIVE/DEAD staining and measurements of lactate dehydrogenase activity; and TUNEL- and ELISA-based quantification of DNA fragments, respectively. Cell migration and proliferation were explored using docetaxel and mitomycin C; temporal and spatial changes in proliferation were assessed by confocal immunofluorescence of proliferating cell nuclear antigen. Gene expression was measured by qPCR.

**Results:**

Photocoagulation of ARPE-19 resulted in denaturation of proteins and reproducible lesion formation. A transient peak in necrosis, followed by a peak in apoptosis was observed in cells within the lesions at 6h and 24h, respectively after photocoagulation. Cell proliferation was depressed during the first hours after photocoagulation, back to control levels at 24h and augmented in the following days. These effects were not limited to cells in the lesions, but also evident in neighbouring cells. Changes in cell proliferation during lesion repair were preceded by changes in cell migration. Altered mRNA expression of genes previously implicated in the regulation of cell proliferation (FOS, IL-1β, IL-8, HMGA2), migration and tissue repairing (TGFBR2, ADAMTS6, TIMP3, CTGF) was observed, as well as increased expression of the alarmin IL33 and the cytoprotective gene HSPA6.

**Conclusions:**

Using a laser system and experimental settings that comply with standards used in clinical practice, we have established a suitable model for in vitro photocoagulation of human RPE cells to isolate their contribution to the beneficial effects of laser treatment.

## Introduction

Diabetic retinopathy is the leading cause of vision loss in working-aged people in developed countries [1] due to vascular leakage in the central part of the retina (macular oedema) and/or ischaemia and subsequent retinal angiogenesis (proliferative retinopathy) [2]. Despite the emerge of new pharmacotherapies, like e.g. intravitreal injections of antibodies against vascular endothelial growth factor [3], photocoagulation remains the golden standard treatment for diabetic retinopathy.

Focal macular oedema is treated by direct laser irradiation of the leaking microaneurysms, while diffuse oedema is treated in a grid pattern over the oedematous part of the retina. Proliferative diabetic retinopathy instead is treated with panretinal photocoagulation, i.e., the retina is exposed to laser irradiation outside the central part, from the outer border of the vessel arcades towards the equator. All three modalities of photocoagulation are pigment-dependent, so the laser light is absorbed by pigmented molecules and converted to heat. In focal photocoagulation this energy conversion takes place in the haemoglobin of the blood, leading to thrombus formation and contraction of the vessel wall [4]. In the grid technique and in panretinal photocoagulation, the energy conversion takes place in the melanin in the retinal pigment epithelium (RPE). Even though it is easier to understand the beneficial effect of focal photocoagulation, where vascular leakage is directly targeted, the molecular mechanisms leading to decreased vascular leakage and proliferation after grid or pan-retinal laser irradiation are still ill defined. This is in part due to the lack of *in vitro* models that mimic the effects of laser irradiation and to difficulties in dissecting the contribution of different cell types in the retina to these processes.

Therefore, we have established a model for *in vitro* photocoagulation of RPE cells, which due to their melanin content are the primary site of laser energy absorption *in vivo*. These cells form a monolayer between the photoreceptor outer segments and choriocapillaris, playing a critical role in the maintenance of visual function [5]. RPE cells transport toxic waste products from the highly metabolic photoreceptors to the choriocapillaris, while supplying them with nutrients. RPE cells are also a source of angiogenic and anti-angiogenic substances and therefore important regulators of retinal vessel formation [5].

In this study, we establish an *in vitro* model of photocoagulation which replicates the changes in cellular necrosis, apoptosis, migration and proliferation observed early after laser irradiation. We also show changes in the expression of genes involved in the regulation of cell proliferation, migration and tissue repairing, as well as the induction of cytoprotective genes. We postulate that this *in vitro* model can be used to further dissect the molecular mechanisms triggered by laser irradiation and the contribution of RPE cells to the process.

## Methods

### Cell Culture

The human RPE cell line ARPE-19 (the American Type Culture Collection, Manassas, VA, USA) was used for all experiments [6]. RPE cells were cultured in DMEM (Invitrogen Ltd, Paisley, UK) containing 100 mg/dL D-Glucose, Sodium Pyruvate, without L-Glutamine and Phenol Red, supplemented with GlutaMAX-I (L-Alanyl-L-Glutamine; Invitrogen) at a concentration of 4 mM, 10% FBS, Streptomycin 100 µg/ml and Penicillin 100 U/ml (Invitrogen). Cells were incubated in humidified environment containing 5% CO_2_ at 37°C and medium changed every third day, reaching a final density of approximately 3×10^6^ cells per cell culture flask within seven days. For all experiments RPE cells were washed once with PBS (pH 7.4±0.05, Invitrogen) and detached from the culture flasks by treatment with 0.05% trypsin-EDTA (Invitrogen). The detached cells were plated at a density of 3×10^4^ cells in 500 µl of medium on glass cover slips (12 mm in diameter, 0.15 mm in thickness) and placed in cell culture wells (16 mm in diameter). The cell culture reached confluency (∼1×10^5^ cells per cover slip) and formed a polarized monolayer 7 days after they were plated (referred to as time “zero”), at which time laser treatment was performed.

### *In vitro* Photocoagulation Model

During the photocoagulation procedure, the cover slips with ARPE-19 cells were temporarily moved to wells without culture medium and placed on top of a black paper to facilitate absorption of the laser energy, as ARPE-19 cells in culture lack pigment. The black paper had been soaked in medium for 2 h prior photocoagulation to create a thin liquid film between the paper and the cover slips, facilitating more uniform heat conduction. Photocoagulation of the confluent RPE cells was accomplished with a frequency-doubled Nd:YAG laser (Visulas 532, Carl Zeiss, Oberkochen, Germany). Each 12 mm cover slip was subjected to 50 evenly spaced laser shots to obtain a similar distribution pattern as that of pan-retinal photocoagulation. Various laser power intensities (200–300 mW) and spot sizes (100–300 µm) were tested in order to determine the settings that yielded higher reproducibility in terms of lesion size and morphology. Laser irradiation time was 0.1 s regardless the setting. Fresh complete medium was added after photocoagulation and the cells were returned to the CO_2_ incubator.

### Morphology

ARPE-19 cells were washed once with PBS and fixed with HistoChoice (Amresco Inc., Solon, OH, USA) at 0h, 30 min, 2h, 6h, 12h, 24h, 48h, 72h and 168h (seven days) after laser treatment. Cells were stained with Haematoxylin (Scharlab S.L., Barcelona, Spain) and Eosin (H & E; Histolab AB, Gothenburg, Sweden) according to the manufacturer’s instructions, inspected on a Nikon Eclipse E800 microscope (Nikon, Tokyo, Japan) and imaged using a Nikon DS-5Mc camera and control unit (Nikon DS-U1) and NIS-Elements version 3.22 (Nikon) software.

### Cell Death

For visualization and discrimination of live and dead ARPE-19 cells after photocoagulation, a LIVE/DEAD Viability/Cytotoxicity Assay Kit was used (Molecular Probes, Eugene, OR, USA). Briefly, cells were washed with PBS before simultaneous staining with green-fluorescent calcein-AM to indicate intracellular esterase activity and red-fluorescent ethidium homodimer-1 to indicate loss of plasma membrane integrity. Ubiquitous intracellular esterase activity and an intact plasma membrane are characteristic of live cells. Both dyes were used at 1µM and cells were incubated for 45 min. Cells were inspected on a Nikon Eclipse E800 microscope using the fluorescence mode with appropriate filter sets, and imaged at 0h, 30 min, 2h, 6h, 12h, 24h, 48h, 72h and 168h (seven days) after laser treatment. ARPE-19 cell death was also measured by quantification of lactate dehydrogenase (LDH) activity in the culture medium using a Cytotoxicity Detection Kit (Roche Applied Science) according to the manufacturer’s instructions.

For visualization of apoptotic ARPE-19 cells, the In Situ Cell Death Detection Kit, POD (Roche Applied Science, Mannheim, Germany) was used according to the manufacturer’s instructions. This assay is based on labeling of DNA strand breaks (TUNEL technology) and therefore, preferentially detects apoptosis vs. necrosis. Cells were washed with PBS before fixation with HistoChoice and imaged at 2h, 6h, 12h, 24h, 48h, 72h and 168h after photocoagulation. For quantification of apoptosis, cytoplasmic histone-associated DNA fragments were measured in cell homogenates and in culture media using the Cell Death Detection ELISA^plus^ assay (Roche Applied Science) at 0h, 2h, 6h, 12h, 24h, 48h, 72h and 168h after photocoagulation according to the manufacturer’s instructions. Briefly, at the different time-points, 100 µl culture medium was removed and placed on ice for 30 min while the cells were lysed. Both the culture medium and the cell lysate were analyzed. An enrichment factor was calculated as the mean absorbance ratio between the photocoagulated samples and the corresponding non-irradiated controls. Six to 8 samples were analyzed for each time-point.

### Cell Proliferation and Migration

**Figure 1:**
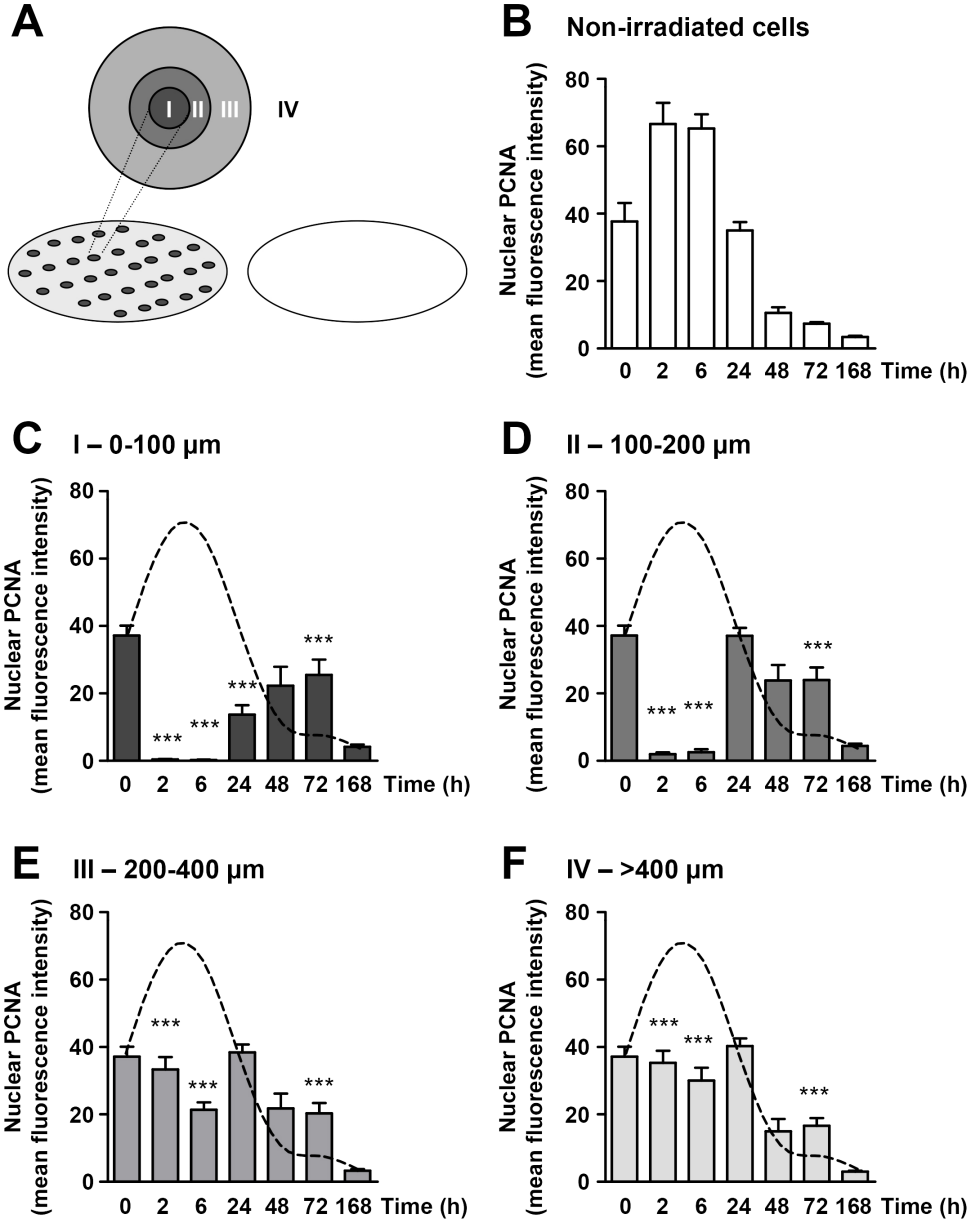
Changes in cell proliferation are not limited to the irradiated cells. Summarized data from confocal immunofluorescence experiments showing changes in nuclear PCNA (proliferating cell nuclear antigen) expression in ARPE-19 cells at various time-points after *in vitro* photocoagulation and under non-irradiated control conditions. **A)** Schematic picture showing a coverslip that has been laser irradiated (dark grey spots) to the left and a control non-irradiated coverslip to the right. The inset on the laser irradiated coverslip shows arbitrary regions within which PCNA levels were quantified, with A being the region that was directly hit by the laser (dark grey) and B–D regions located at increasing distance from the centre of the spots (medium grey, light grey and white, respectively). **B)** Summarized data showing PCNA fluorescence intensity in non-treated control cells at the time points indicated. **C–F)** Summarized data showing PCNA fluorescence intensity in region I (0–100 µm radius), II (100–200 µm radius), III (200–400 µm radius) and IV (>400 µm radius) at the time points indicated. The dashed lines represent a cubic spline curve fit of the PCNA levels in non-irradiated control cells showed in panel A; shown for presentation purposes. *** *p*<0.001 vs non-treated cells at each time-point.

ARPE-19 cells were fixed in ice cold methanol at 0h and at 2h, 6h, 24h, 48h, 72h and 168h after photocoagulation. Immunofluorescence experiments were performed using a monoclonal antibody against PCNA (Proliferating Cell Nuclear Antigen, PC10, Santa Cruz Biotechnology, Santa Cruz, CA, USA) and a secondary antibody, Cy5 donkey-anti-mouse IgG (1∶300, Jackson ImmunoResearch, Suffolk, U.K.). Nuclear regions and individual cells were identified using SYTOX Green (1∶3,000, Molecular Probes, Eugene, OR, USA). Three coverslips were examined for each time point at x10 (0.30 numerical aperture) in a Zeiss LSM 5 Pascal laser scanning confocal microscope (Carl Zeiss, Oberkochen, Germany) and nuclear PCNA intensity was quantified using the Zeiss LSM 5 analysis software. For laser-treated cells, 13–33 laser spots were imaged for each time point. Four to six boxes were positioned at defined distances from the lesion centre to quantify nuclear PCNA intensity in different regions (Fig. 1A). For non-laser treated control cells, 3–7 images were obtained from each coverslip.

For discrimination between cell proliferation and migration during repair of the laser lesions, we first determined the non-toxic concentration of docetaxel (Sigma Aldrich, Stockholm, Sweden) that effectively blocks migration without affecting proliferation and the non-toxic concentration of mitomycin C (Sigma Aldrich) that effectively blocks proliferation without affecting migration. For cell proliferation, ARPE-19 cells were plated as explained above under “Cell culture” at a density of 3×10^4^ cells and cultured for 7 days until they reached confluency, at which time (0 h) they were incubated with docetaxel (0.001–10 nM) or mitomycin C (0.1–10 µM) for 72 hours. After treatment, cells were trypsinized and live cells were counted using a Bürker chamber after exclusion of Trypan Blue (Sigma Aldrich) positive cells. Data is expressed as percentage growth from treatment start (0 h). Cell viability was also assessed using a CellTiter 96 AQueous One Solution Cell Proliferation Assay Reagent (Promega, Stockholm, Sweden) according to the manufacturer’s instructions. This assay is based on the spectrophotometric detection of a colored formazan product converted from an (3-(4,5-dimethylthiazol-2-yl)-5-(3-carboxymethoxyphenyl)-2-(4-sulfophenyl)-2H-tetrazolium) (MTS) compound by NADPH or NADH via metabolically active cells. After culture, fresh culture medium was added together with CellTiter 96 Aqueous One Solution Cell Proliferation Assay Reagent and incubated for 1 h before measuring absorbance at 490 nm with a 96-well plate reader. For the effects of the drugs on cell migration, confluent ARPE-19 cells on 12 mm cover slips were scratched with a sterile pipette tip and photographed just after scratch (0 h) and 24 h later after incubation with or without docetaxel or mitomycin C at various concentrations. Cells were inspected on a Nikon TMS microscope and imaged using a Nikon DS-Fi1 camera and NIS-Elements F version 2.20 software. Scratch areas were measured using Image J and data were expressed as percentage of scratch area closed after 24 h. The differential effects of docetaxel and mitomycin C on migration and proliferation during the repair of laser lesions were then evaluated by measurement of the size of the cell-free region at the center of the lesions 12, 24, 48 and 72 h after photocoagulation.

### RNA Extraction

RNA extraction was performed using Nucleospin RNA XS (Machery-Nagel, Düren, Germany) at 6h and 24h after photocoagulation, according to the manufacturer’s description. RNA quality and concentration was assessed using the NanoDrop 1000 Spectrophotometer (Thermo Fisher Scientific, Wilmington, DE, USA). RNA samples were stored at –80°C until further analysis.

### Quantitative RT-PCR (qPCR)

cDNA was synthesized from RNA using the RevertAid First Strand cDNA Synthesis Kit (Fermentas, St. Leon-Rot, Germany). mRNA levels were analyzed with the real-time RT-PCR 7900HT system (Applied Biosystems, Foster City, CA, USA) using 10 µl TaqMan Gene Expression Master Mix (Applied Biosystems), 1 µl TaqMan Gene Expression Assay, 1 µl cDNA and 8 µl deionized, diethylpyrocarbonate (DEPC) treated water (Fermentas). All assays were from Applied Biosystems: *HSPA6* Heat shock 70kDa protein 6 (Hs00275682_s1); *CTGF* Connective tissue growth factor (Hs00170014_m1); *FOS* FBJ murine osteosarcoma viral oncogene homolog (Hs00170630_m1); *HMGA2* High mobility group AT-hook 2 (Hs00971724_m1); *IL33* Interleukin 33 (Hs01125943_m1); *IL1B* Interleukin 1, beta (Hs01555410_m1); *ADAMTS6* ADAM metallopeptidase with thrombospondin type 1 motif, 6 (Hs01058097_m1); *IL8* Interleukin 8 (Hs00174103_m1); *TGFBR2* Transforming growth factor, beta receptor II (70/80kDa) (Hs00234253_m1); *ANKRD1* Ankyrin repeat domain 1 (cardiac muscle) (Hs00173317_m1); *TIMP3* TIMP metallopeptidase inhibitor 3 (Hs00165949_m1). Cyclophilin B (Hs01018502_m1) was used as endogenous control. The relative quantity of the target gene was calculated using the comparative threshold method (ΔΔCt). If the standard deviation of the duplicate Ct value differed more than 0.16, the sample was rerun.

### Statistics

Kruskal-Wallis and Mann-Whitney U test were used to test for significance. *P* values <0.05, <0.01 and <0.001 were used after Bonferroni correction for multiple comparisons. Statistical calculations were performed on SPSS software v 20 (IBM, Armonk, NY, USA). Data is presented as mean ±SEM in all graphs.

## Results

### Photocoagulation Settings

**Figure 2:**
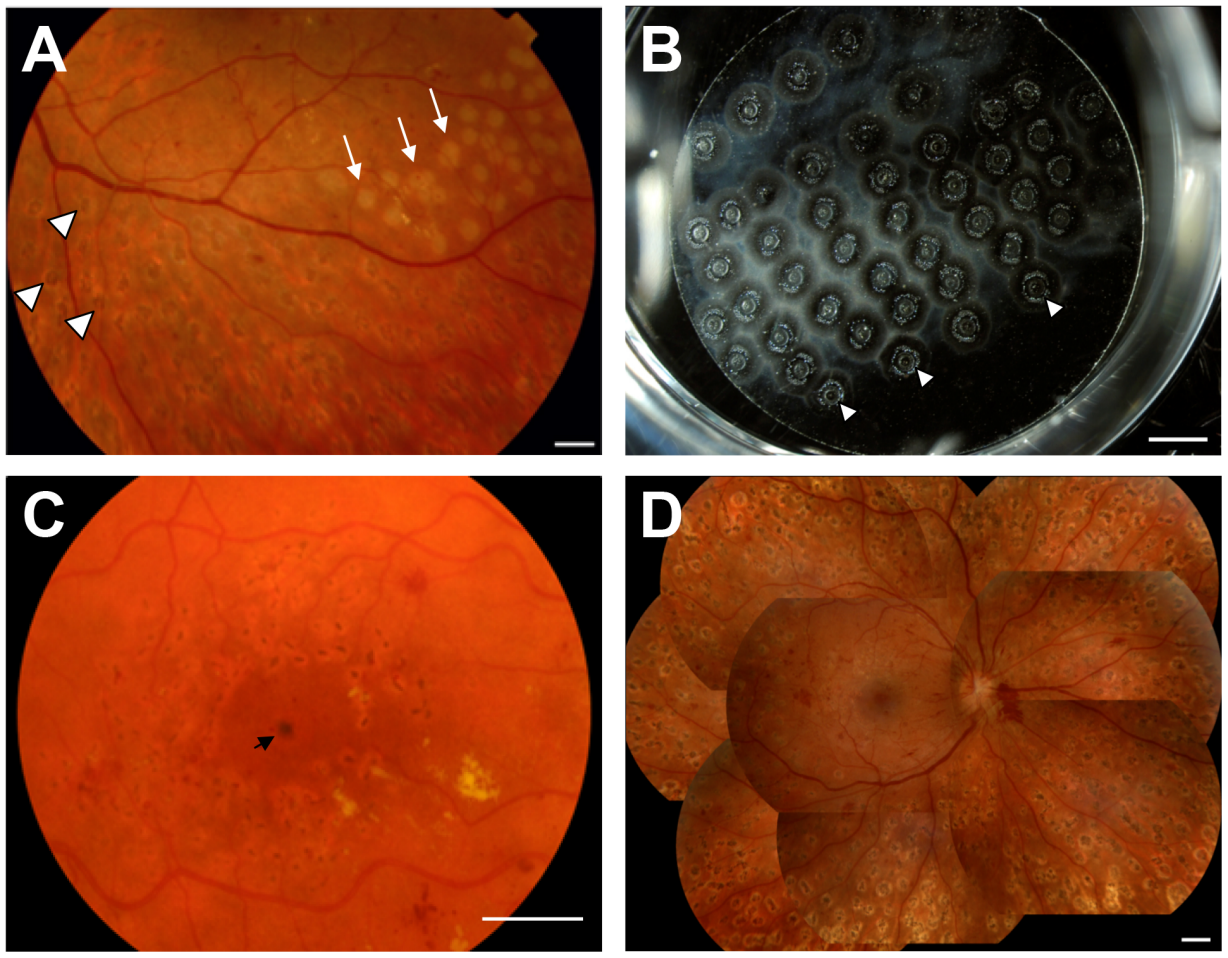
In vivo and in vitro photocoagulation. Representative images showing: **A)** Pale dots (white arrows) in the upper right portion of the field observed shortly after exposure to the laser (spot size = 500 µm). The image was acquired during a second session of pan-retinal photocoagulation 10 minutes after laser exposure in a patient with proliferative diabetic retinopathy. The darker pigmented dots (arrowheads) observed in the lower part of the field are the result of a previous photocoagulation session 13 days earlier. **B)** Lesions observed minutes after *in vitro* photocoagulation of ARPE-19 cells cultured on glass cover slips (spot size = 200 µm). Images were obtained using a Nikon D2X digital camera. Small gas bubbles appear between the paper and the glass cover slip after photocoagulation, forming the pale punctuated rings observed around the lesions. Small bubbles often merge into bigger central bubbles (white arrowheads). **C)** Pigmentation after grid photocoagulation in a patient with diabetic macular oedema. **D)** Pigmentation after pan-retinal photocoagulation in a patient with proliferative diabetic retinopathy. Scale bars represent 1500 µm.

The immediate clinical appearance of a laser lesion is characterized by a pale discoloration due to denaturation of proteins within the retina and RPE [7], [8], as observed in Fig. 2A. Within a couple of weeks the lesions usually become pigmented as a result of RPE cell accumulation (Fig. 2A, C and D). In order to achieve reproducible *in vitro* lesions with similar size and spacing pattern as those observed *in vivo,* we tested the impact of varying the laser intensity and the spot size. *In vitro*, the highly conductive glass cover slips will favour the lateral transfer of the thermal transients, whereas *in vivo*, these are limited due to the water content of biological samples. To compensate for this difference in conductance, laser spot sizes *in vitro* were smaller than those generally used *in vivo* for pan-retinal photocoagulation (100–300 µm vs. 500 µm). Of all tested experimental laser setting, the combination of 300 mW of laser power, 200 µm spot size and 0.1 s irradiation duration were found to yield the most reproducible lesions (Fig. 2B) and therefore used throughout this study. Fig. S1 shows images of H & E stained ARPE-19 cells 24 h after photocoagulation using all tested laser setting combinations.

As also evidenced in Fig. 2B, the laser leaves a circular impression of the same size of the beam (200 µm) as it hits the pigment source (black paper). Within one minute, numerous small gas bubbles appear between the paper and the glass cover slip, forming the pale punctuated rings observed around the lesions in Fig. 2B. The small bubbles often merge into a bigger central bubble within the next five minutes (white arrowheads in Fig. 2B) and gradually disappear within the following 5–10 minutes. These bubbles are not in contact with the cells but contribute to the artifactual appearance of the *in vitro* lesions in Fig. 2B. The monolayer nature of the *in vitro* system as opposed to the complex multilayered architecture of the intact retina and the fact that cultured cells are temporarily moved to wells without culture medium during photocoagulation may also contribute to the somewhat different appearance of the *in vitro* lesions when compared to *in vivo* lesions in Fig. 2.

### Time Course of Repair

**Figure 3:**
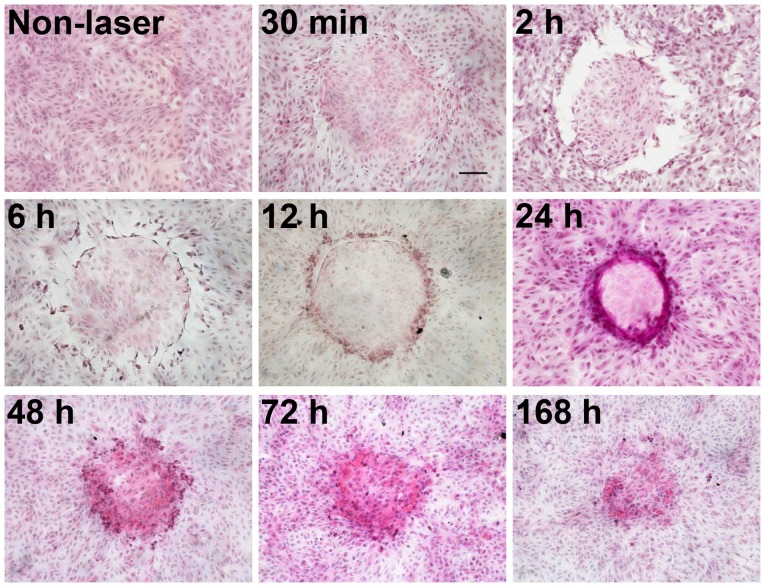
Haematoxylin & Eosin stained ARPE-19 cells at various time points after *in vitro* photocoagulation. The following settings were used: 200 µm, 300 mW, 0.1 s. Scale bar represents 100 µm; magnification 10X. Control cells at time 0 (non-laser) are shown for reference.

Photocoagulation of ARPE-19 cells *in vitro* resulted in visible morphological changes already at 30 min (Fig. 3). An increased turbidity of the H & E staining was observed at the centre of the lesions (mean diameter 456.9±6.3 µm, N = 104 lesions), likely reflecting protein denaturation and the result of the high thermal input received by these cells. Two hours after laser irradiation, cell detachment occurred at the periphery of the lesions, as evidenced by an empty rim in the H & E images. This detachment is probably due to cell damage caused by heat dissipation outwards from the centre of the lesion. The images also highlight that in this experimental model the thermal transients are at least able to travel as far as to the outer edges of the empty rims (i.e. a mean rim thickness of 38.7±4.0 µm, N = 158 lesions). At 6 h and 12 h, these empty areas were smaller due to gradual re-population with migrating cells from outside the lesion. At 24 h, the empty rim was completely covered by cells organized in a densely packed circular ring. At 48 h, the accumulated cells at the circular rim had started to cover the centre of the lesion, while at 72 h this area was almost fully covered by ARPE-19 cells. Seven days after photocoagulation, cells completely covered the lesion areas, but were still organized in a less homogeneous pattern (Fig. 3).

### Necrosis and Apoptosis

**Figure 4:**
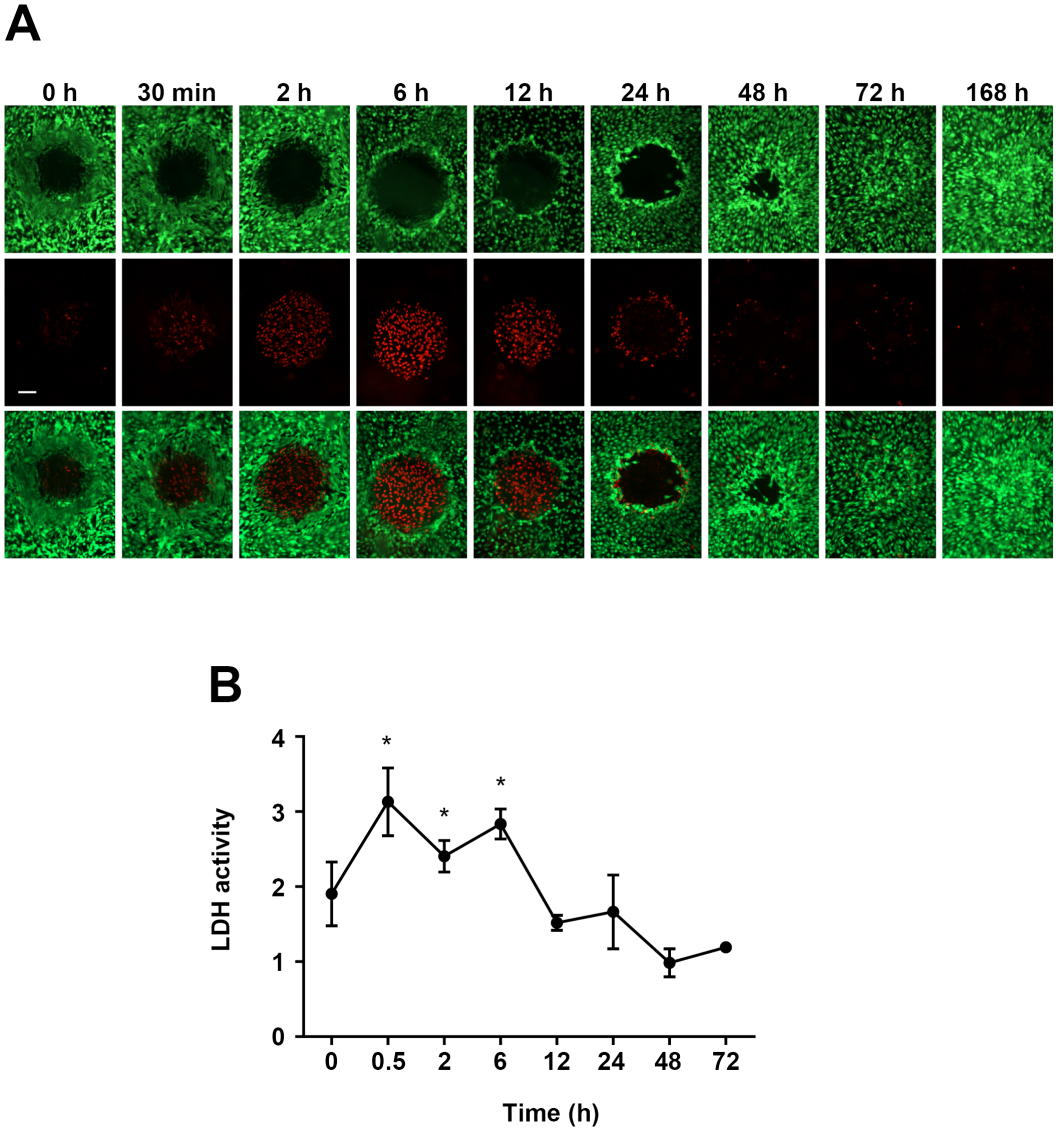
A) Visualization of dead (red) and live (green) ARPE-19 cells after photocoagulation. Loss of plasma membrane integrity results in uptake of red-fluorescent ethidium homodimer-1 in dead cells (middle panels), while living cells stain positive for intracellular esterase activity labelled with green-fluorescent calcein-AM (upper panels). Lower panels show merged images for the two fluorophores. Scale bar represents 100 µm. **B)** Early transient increase in necrosis in ARPE-19 cells, as assessed by quantification of lactate dehydrogenase activity in the culture medium at various time-points after photocoagulation. Data is expressed as mean absorbance ratio between the photocoagulated samples and the corresponding non-irradiated controls for each time-point. Four samples were analyzed at each time-point; *p<0.05 vs non-irradiated controls at 30 min, 2 h and 6 h.

One of the consequences of the laser irradiation is the generation of heat, which per se can induce necrosis through membrane damage or apoptosis through aggregation of toxic denatured proteins or activation of the intrinsic apoptotic pathway [9]. Using a LIVE/DEAD assay based on the uptake of red-fluorescent ethidium homodimer-1 in dead cells due to loss of membrane integrity and on the staining of intracellular esterase activity with green-fluorescent calcein-AM in living cells, we demonstrated that necrotic ARPE-19 cells were clearly restricted to the laser irradiated areas and appear early after photocoagulation, with a peak at 6 hours (Fig. 4A). No dead cells (red) and only live cells (green) were detected in areas in between laser lesions (Fig. S2A) or on cover slips that have not been laser treated (Fig. S2B). As a complementary approach, we also measured the release of LDH to the culture medium at various time-points after photocoagulation and found, in agreement with the fluorescence experiments shown in 3A, that LDH was significantly higher in the medium of laser treated cells compared to untreated controls at 30 min, 2 and 6 h after photocoagulation, but not at later time-points (Fig. 4B).

**Figure 5:**
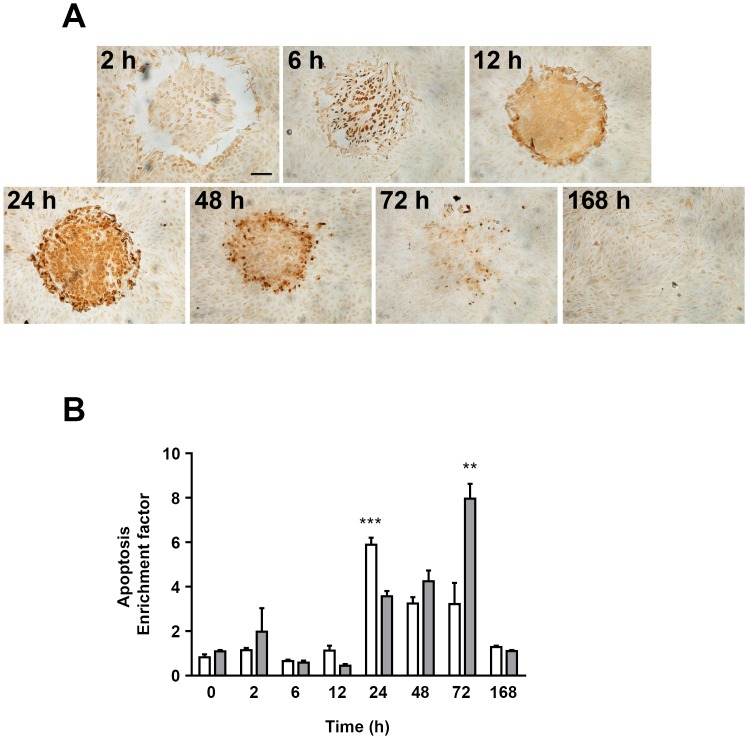
Transient increase in apoptosis in ARPE-19 cells after photocoagulation. **A)** Representative images showing apoptotic ARPE-19 cells visualized by TUNEL-staining (brown) at various time-points after *in vitro* photocoagulation. Scale bar represents 100 µm; magnification 10X. **B)** Summarized data showing measurements of cytoplasmic histone-associated DNA fragments in cell homogenates and culture media at various time-points after *in vitro* photocoagulation. Data is expressed as mean absorbance ratio between the photocoagulated samples and the corresponding non-irradiated controls for each time-point (apoptosis enrichment factor). Six to 8 samples were analyzed for each time-point. ***p<0.001 vs 12 h and vs 48 h in the cell-homogenates; **p<0.01 vs 48 h and vs 168 h in the medium.

Apoptotic ARPE-19 cells were also evident in the laser irradiated areas but these appeared at later time-points exhibiting a maximal intensity 24 h after photocoagulation (Fig. 5A). Using a complementary approach, we measured DNA fragmentation in ARPE-19 cell homogenates and in the culture medium. DNA fragmentation reflecting apoptosis was higher in the samples from laser treated cells when compared to control samples (Fig. 5B). In agreement with the *in situ* apoptosis assay (Fig. 5A), apoptosis levels in the cell homogenates peaked 24 h after photocoagulation (Fig. 5B). The later peak at 72 h measured in the medium was likely due to accumulation of apoptotic DNA fragments released from the cells in the culture media. This is supported by the fact that DNA fragmentation levels in the culture media at 168 h were lower than those measured at 72 h, a time-point after which culture media was routinely exchanged.

### Cell Proliferation and Migration

**Figure 6:**
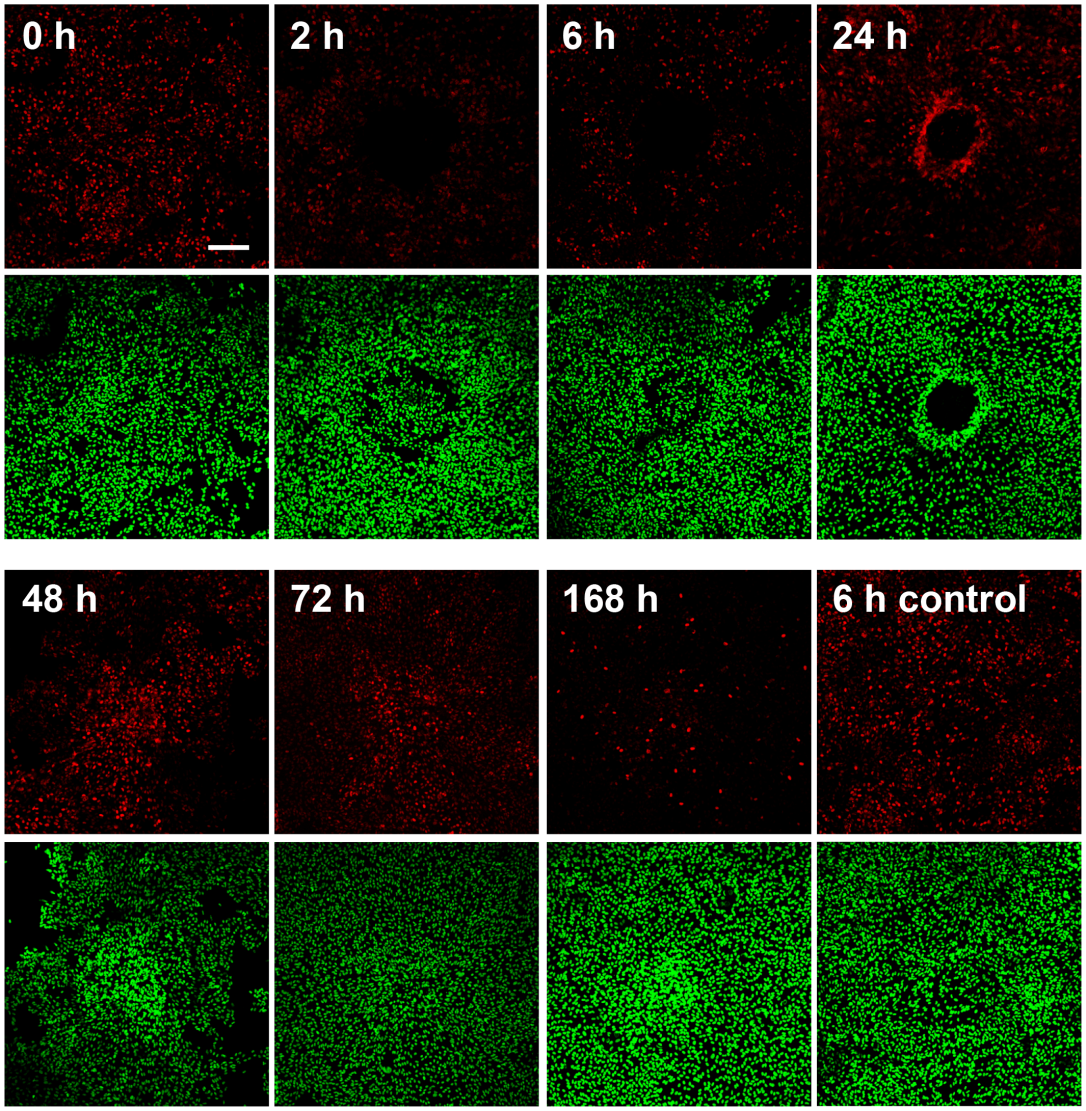
Confocal immunofluorescence images showing PCNA (proliferating cell nuclear antigen) expression (red) in ARPE-19 cells at various time-points after *in vitro* photocoagulation. Nuclei are stained with SYTOX Green. Expression of PCNA in non-irradiated control cells at 6 h is also shown for comparison. Scale bar represents 200 µm.

To study the effect of photocoagulation on cell proliferation, we measured the expression of the S-phase marker PCNA in ARPE-19 cells at various time points after laser irradiation. Using confocal immunofluorescence, we first examined the basal levels of nuclear PCNA in non-irradiated control cells maintained in culture up to 7 days (168 h). In this as in all other experiments, the culture medium was replaced by fresh medium after photocoagulation (0 h) and after 72 hours in culture. As shown in Fig. 1B, proliferation reached a peak at 2–6 h, returned to baseline at 24 h and declined further thereafter. This is in agreement to what others have described, namely that ARPE-19 cells can continue to proliferate despite confluence [10]. In contrast, photocoagulated cells displayed dramatically reduced PCNA levels after 2 h that remained reduced at 6 and 24 h (Fig. 1C; region “I”). This is in part due to lower number of cells in this region due to photocoagulative necrosis as shown in Fig. 4. However, the same was true for cells located further away from the more central region I, with the inhibitory effect decaying with increasing distance but still evident in regions >400 µm away from the centre of the laser spot (Fig. 1D–F; regions “II”, “III” and “IV”), where no necrosis was detected (Fig. 4A and Fig. S2A). This depression of cell proliferation was transient; as evidenced by restored PCNA levels 24–48 hours after irradiation depending on the region examined. Moreover, 72 hours after irradiation, ARPE-19 cells in all regions, exhibited significantly higher PCNA levels than those in control cells. Measurements in areas >600 µm away from the centre of the laser spot were not different from those at >400 µm (not shown). Figure 6 shows original confocal images depicting the changes in PCNA expression at various times after laser irradiation summarized in Fig. 1.

**Figure 7:**
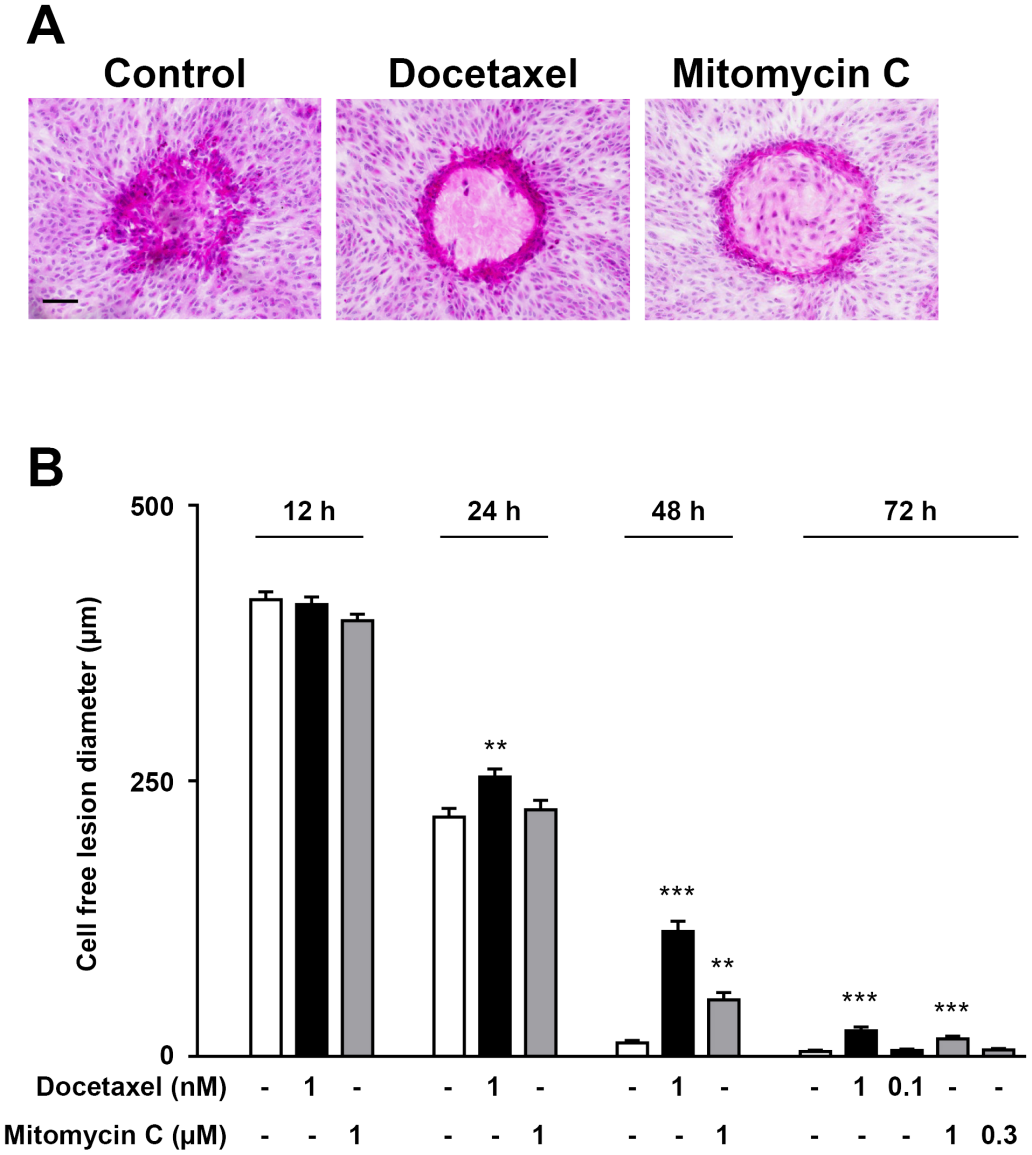
Both cell migration and proliferation contribute to lesion repair. **A)** Representative H&E images showing ARPE-19 cells that had been cultured with and without docetaxel (1 nM) or mitomycin C (1 µM) for 48 h after laser photocoagulation. Scale bar represents 100 µm. **B)** Summarized data from experiments as in A, showing the effects of culture with and without docetaxel (1 nM) or mitomycin C (1 µM) for 12 h, 24 h or 48 h on the diameter of the cell-free region of the lesion. For the 72 h time-point, two concentrations of docetaxel (1.0 and 0.1 nM) and of mitomycin C (1.0 and 0.3 µM) were tested. Four samples were analyzed for each condition or time-point; ***p<0.001 and **p<0.01 vs. untreated controls for the corresponding time-points.

It is well recognized that the repair of the laser lesion involves not only cell proliferation, but also migration of the adjacent RPE cells. To discriminate between these two processes, we used docetaxel to block cell migration or mitomycin C to block cell proliferation and compared the size of the cell free areas of the laser lesions to untreated controls at 12, 24, 48 and 72 h after photocoagulation (Fig. 7). In order to determine non-toxic concentrations of docetaxel that effectively block migration without affecting proliferation and of mitomycin C that effectively block proliferation without affecting migration, dose-response experiments were performed prior photocoagulation experiments (Fig. S3). Using an *in vitro* scratch assay, we found that both 1 and 10 nM docetaxel effectively compromised cell migration as evidenced by significantly reduced scratch area covered with cells 24 h after wounding (Fig. S3A). Given that 10 nM was found to cause cytotoxicity as determined using the MTS assay (data not shown), docetaxel was used at 1 nM. Mitomycin C was found to limit cell proliferation at all doses tested (Fig. S3C), but 0.3 and 1 µM were used based on the lack of cytotoxic effects or effect of these doses on cell migration (Fig. S3B). Using these previously titrated doses, we found that both docetaxel and mitomycin C effectively delayed the repair process of the laser lesions (Fig. 7A). Inhibition of cell migration with docetaxel resulted in larger cell-free areas in the center of the lesions than non-treated controls and this difference was noticeable 24 h after photocoagulation (Fig. 7B). Inhibition of cell proliferation on the other hand, slowed down the repair process but at a later time-point (48 h, Fig. 7B), suggesting that changes in cell migration may precede cell proliferation during the repair process after laser photocoagulation.

### qPCR

**Figure 8:**
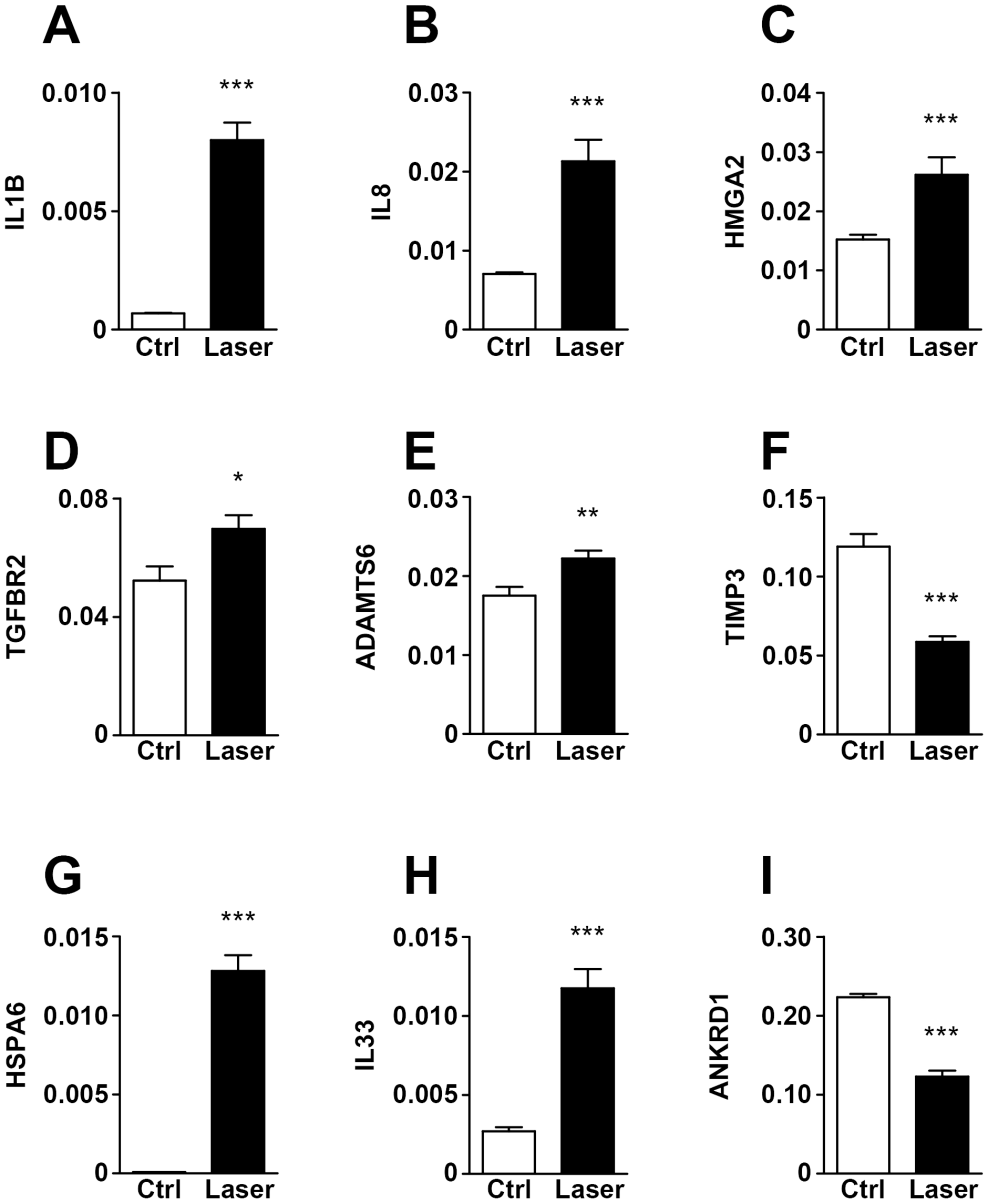
Photocoagulation results in changes in gene expression. *IL1β, IL8, HMGA2, TGFBR2, ADAMTS6, TIMP3, HSPA6, IL33* and *ANKRD1* mRNA expression in ARPE-19 cells 24 h after photocoagulation and in control non-irradiated cells. Measurements were performed by qPCR using cyclophilin B as an endogenous control. ***p<0.001; **p<0.01 and *p<0.05 vs. control; *n*≥6.

Several genes known to promote cell proliferation were significantly up-regulated 24 h after photocoagulation, such as the cytokines IL1β, IL8 and the oncogene and regulator of pluripotency in stem cells HMGA2 [11]. Production of both cytokines has been demonstrated in RPE cells, but the HMGA2 finding is novel. Six hours after photocoagulation FOS was significantly up-regulated, a target known to promote proliferation in RPE cells via cyclin D1 expression [12] (Figure S4). We also found increased mRNA expression of the TGFβ receptor 2 (TGFBR2), of the matrix metalloprotease ADAMTS6, known to be up regulated in ARPE-19 cells upon TNFα stimulation [13] and CTGF, as well as decreased expression of TIMP3, recently established as a signature gene of potential role in AMD pathogenesis [14] all consistent with enhanced tissue remodeling capacity important for lesion repairing. ANKRD1, a co-activator of p53 and pro-apoptotic gene was down regulated at 24 h. The cytokine *IL33* which is proposed to be an alarmin released from necrotic cells [15] and heat shock 70 kDa protein 6 (HSPA6), an inducible heat shock protein known to increase stress tolerance and survival [16] were readily induced by *in vitro* photocoagulation in ARPE-19 cells. The induction was clear 6 h after the laser irradiation and still significant at 24 h (Fig. 8 and Figure S4).

## Discussion

In this study, we have established a method for *in vitro* photocoagulation of human RPE cells which results in reproducible lesions of similar size and spatial distribution to the ones observed *in vivo* short after laser irradiation. In this model we have demonstrated an initial induction of necrosis (∼6 h) followed by apoptosis (∼24 h) only in the laser irradiated RPE cells. We also show that the repair of the laser lesions involves both cell migration and proliferation, but that changes in cell migration seem to precede the changes in cell proliferation. Interestingly, the changes in cell proliferation were not limited to the lesions. Also, photocoagulation of RPE cells resulted in changes in the expression of genes involved in the regulation of cell proliferation, migration and tissue repairing, as well as the induction of the alarmin *IL33* and cytoprotective heat shock protein *HSPA6*. We believe that this is a suitable model to study the role of RPE cells in the processes that occur after laser coagulation and to investigate their potential contribution to the beneficial effects observed in clinical practice.

Laser-tissue interactions are affected by the wavelength, energy delivered, spot size and duration of application [4]. The parameters used in our experimental setup (300 mW of power, 200 µm spot size and 0.1 s duration, with 50 spots evenly distributed per 12 mm cover slip) were found to be optimal to obtain reproducible spots. Previous studies have used similar approaches to induce laser photocoagulation in cultured RPE cells [17]–[21], however the nature of the laser (i.e. diode lasers, less often used for retinal vascular disease since they cause pain [4]) and experimental settings (i.e. smaller (50 µm) or larger (625 µm) spot sizes) used in these studies deviated to a larger extent from standards use in clinical practice. The strength of our model is the use of a laser system currently also applied for treatment of patients and experimental settings that comply to the standards established by the Early Treatment Diabetic Retinopathy Study [22], including 0.1 s exposures and spot sizes ranging from 100–500 µm. Also, we tried to obtain a similar spot spacing *in vitro* as that normally required for panretinal photocoagulation, since the pattern density of the lesions has been suggested to have an impact on clinical efficacy [23].

Apoptosis of RPE cells is a not only an important event in the pathogenesis of age-related macular degeneration [24], but also the result of high-glucose induced oxidative damage in diabetic retinopathy [25]. Given that RPE cells are essential for the support of photoreceptor function; efforts have focused on identifying mechanisms to prevent apoptosis in an attempt to maintain visual function. Our results shows that laser photocoagulation has indeed a negative impact on RPE cell integrity by virtue of increased photocoagulative necrosis and apoptosis, but this effect is transient and limited to the fraction of cells at the centre of the lesions and exposed to the highest thermal input. This is in line with the study of Barak et al [21] who demonstrated an increase in apoptosis in RPE cells hours after diode laser irradiation. The fact that RPE cells underwent apoptosis relatively late after photocoagulation would suggest a mechanism triggered by the toxic accumulation of aggregated proteins typical of cells with high heat tolerance, as opposed to a mechanism dependent on MAPK signalling and the intrinsic apoptotic pathway characteristic of cells with low heat tolerance [9]. In any case, the loss of apoptotic RPE cells seems to be compensated by an increased proliferative capacity after laser irradiation (Figure 1). The overall decrease expression of ANKRD1 mRNA at 24 hours may also contribute to limiting the apoptotic response.

Short after *in vivo* photocoagulation, lesions usually become pigmented as a result of RPE cell accumulation, as shown in Fig. 2A, C and D. This has been attributed, at least in animal models to changes in RPE cell proliferation and migration [26], [27]. Using our *in vitro* model, we could temporally and spatially dissect the changes in RPE migration and proliferation induced by laser coagulation. While the proliferative capacity of RPE cells was depressed during the first hours after photocoagulation, cells recovered and were back to control levels already 24 hours after laser irradiation to then exhibit significantly higher proliferating rates during the following days. Interestingly, this pattern was not exclusive to the lesion cells, but also a feature of neighbouring cells, suggesting thermal spread of the laser beyond the lesion area. Both theoretical and experimental data show that the temperature decreases rapidly with increasing distance from the lesion and that the spread of the retinal damage would be negligible 500 µm from the lesion [28]. In our model in which a higher thermal conductance can be anticipated, the central zone of the lesions where the protein denaturation occur and the periphery, where cell detachment and empty rims were observed averaged 456 µm in diameter. This zone is clearly affected by the thermal transient, but effects on cells outside this zone, although less severe, may still be possible. The fact that the effects in proliferation decayed with increasing distance from the lesion and were significant in areas outside the lesions (>600 µm), would support this idea. However, it is also possible that the changes outside the lesions may not be solely induced by heat, but also mediated by a diffusible factor from RPE cells upon laser irradiation. A potential candidate is pigment-epithelium-derived factor (PEDF), which is up regulated in human retinal pigment epithelial cells after photocoagulation [20] and has been shown to control RPE proliferation [29].

Another growth factor that has been shown to be secreted by RPE cells upon laser photocoagulation is transforming growth factor-beta (TGFβ) 2 [17] and important regulator of cell migration. The here described increased expression of *TGFBR2* may suggest potentially enhanced signaling via TGFβ in this context. Expression results indicate that several important mediators of cell proliferation and tissue remodeling are engaged upon photocoagulation in this model, mediators that can be predicted to play a role in the lesion repairing process and in maintaining RPE integrity. Another observation was the induction of *HSPA6* mRNA, normally not expressed in untreated RPE cells. The induction of heat shock proteins is a well-described effect of the elevated temperature upon laser irradiation is [30] and critical for orchestrating a cytoprotective response against severe cellular stress [31], [32]. Interestingly, *IL33*, a novel cytokine of the IL1 family involved in the polarization of T cells towards T helper 2 cell phenotype was also increased. IL33 is proposed to be released from necrotic cells as an alarmin and very recently, it was suggested to play a role in the pathogenesis of AMD [33].

In conclusion, further research will be required in order to understand the sequence of biological events triggered by laser photocoagulation leading to reduced risk of vision loss from retinal vascular disease. Our simplified *in vitro* model lacks potential influences from other surrounding cells types also affected by the laser, but allows a better characterization of the contribution of RPE cells to the process.

## Supporting Information

Figure S1 Lesion morphology is affected by laser beam size and intensity. H&E stained ARPE-19 cells imaged 24 h after *in vitro* photocoagulation for 0.1 s. Images show the effect of changing the laser beam size and intensity: **A)** 100 µm and 200 mW; **B)** 200 µm and 200 mW; **C)** 300 µm and 200 mW; **D)** 200 µm and 300 mW. Scale bar represents 100 µm; magnification 10X. The most reproducible laser lesions were induced with laser settings as in D and were therefore used throughout the manuscript.
(TIF)

Figure S2 Cell death is not detected in control non-photocoagulated ARPE-19 cell preparations or in cells surrounding the laser lesions. Simultaneous staining with green-fluorescent calcein-AM (left panels) and red-fluorescent ethidium homodimer-1 (middle panels) to discriminate between live and dead cells demonstrated absence of dead cells (red) and only live cells (green) in **A)** areas in between laser lesions 0 and 24 h after photocoagulation; and in **B**) control cells on cover slips that have not been laser treated. Right panels show merged images for the two fluorophores. Scale bar represents 100 µm.
(TIF)

Figure S3 **A and B)** Effect of docetaxel (A) and mitomycin C (B) at various concentrations on migration, as determined by a scratch wound assay. Migration was determined as the % scratch area closed 24 h after wounding. Seven to 9 samples were analyzed for each treatment. **C)** Dose-dependent effect of docetaxel and mitomycin C on cell proliferation. Cells were cultured on glass cover slips and counted 72 h after the start of the treatment. Three to 6 samples per treatment were analyzed. **p<0.01 vs. untreated cells.
(TIF)

Figure S4 Photocoagulation results in changes in gene expression. FOS, CTGF, IL33 and HSPA6 mRNA expression in ARPE-19 cells 6 h after photocoagulation and in control non-irradiated cells. Measurements were performed by qPCR using cyclophilin B as an endogenous control. ***p<0.001 and **p<0.01 vs. control; *n*≥6.
(TIF)
