# Supplementary figures and images for: Photocoagulation of Human Retinal Pigment Epithelial Cells In Vitro: Evaluation of Necrosis, Apoptosis, Cell Migration, Cell Proliferation and Expression of Tissue Repairing and Cytoprotective Genes
Source: PLoS One. 2013 Aug 1;8(8):e70465. doi: 10.1371/journal.pone.0070465 (PMC3731268; doi:10.1371/journal.pone.0070465)

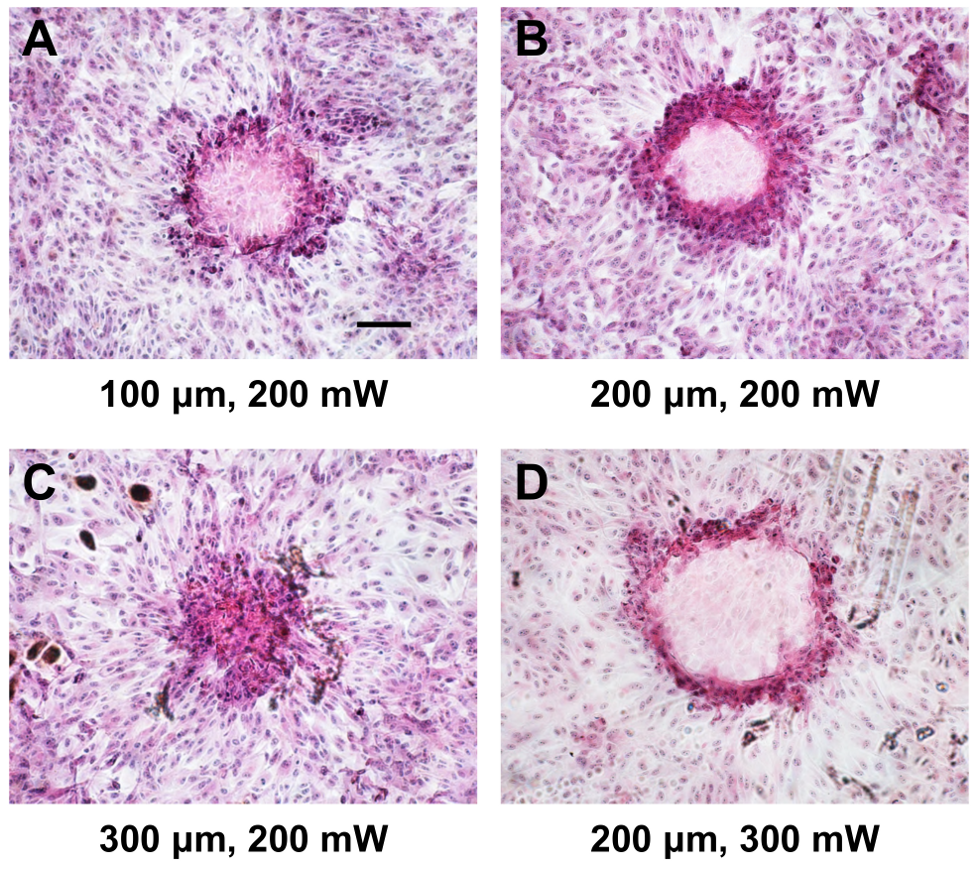

Supplement: Figure S1 — Lesion morphology is affected by laser beam size and intensity. H&E stained ARPE-19 cells imaged 24 h after in vitro photocoagulation for 0.1 s. Images show the effect of changing the laser beam size and intensity: A) 100 µm and 200 mW; B) 200 µm and 200 mW; C) 300 µm and 200 mW; D) 200 µm and 300 mW. Scale bar represents 100 µm; magnification 10X. The most reproducible laser lesions were induced with laser settings as in D and were therefore used throughout the manuscript. (TIF) [file pone.0070465.s001.tif]

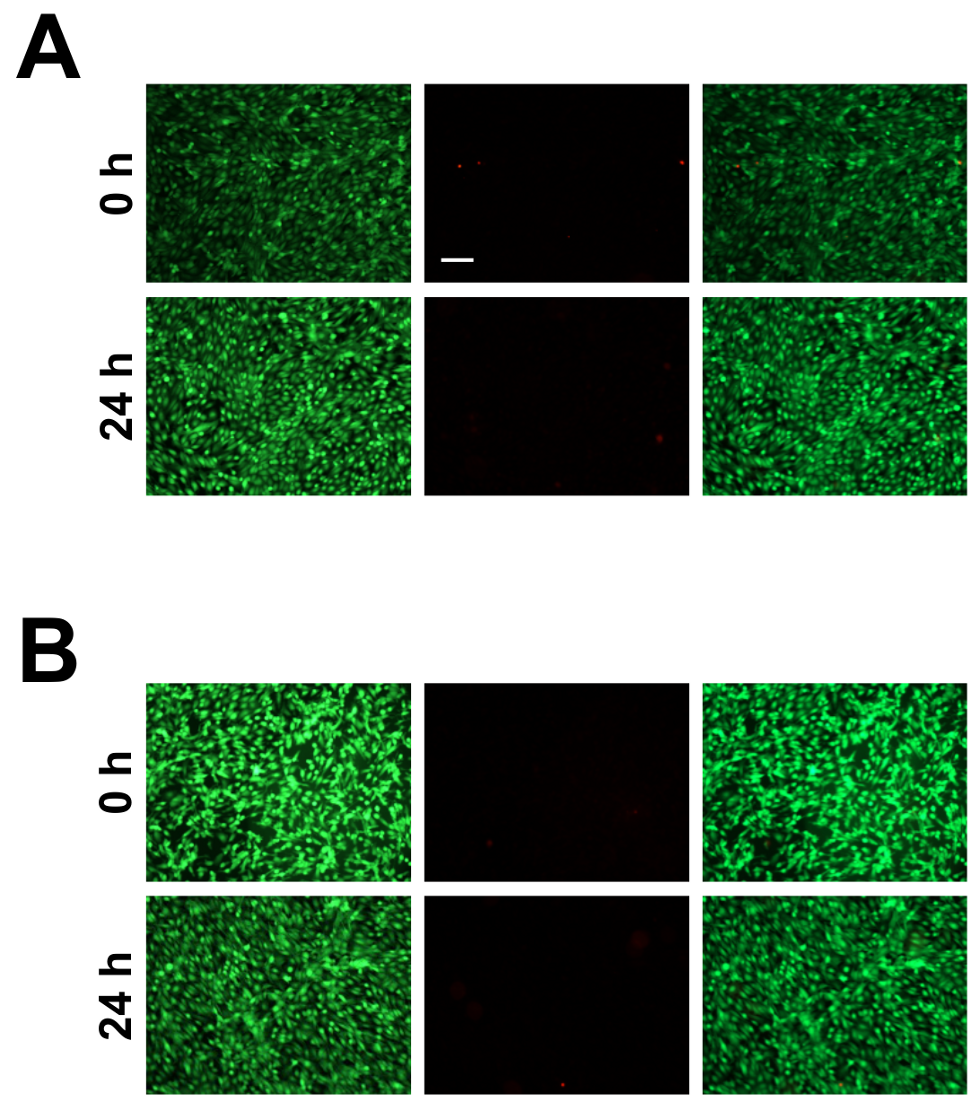

Supplement: Figure S2 — Cell death is not detected in control non-photocoagulated ARPE-19 cell preparations or in cells surrounding the laser lesions. Simultaneous staining with green-fluorescent calcein-AM (left panels) and red-fluorescent ethidium homodimer-1 (middle panels) to discriminate between live and dead cells demonstrated absence of dead cells (red) and only live cells (green) in A) areas in between laser lesions 0 and 24 h after photocoagulation; and in B) control cells on cover slips that have not been laser treated. Right panels show merged images for the two fluorophores. Scale bar represents 100 µm. (TIF) [file pone.0070465.s002.tif]
